# Generative Artificial Intelligence as a First Responder in Adolescent Testicular Torsion: A Case Report

**DOI:** 10.7759/cureus.109257

**Published:** 2026-05-20

**Authors:** Ibraheem Alami, Qasam Soboh, Haya Taha, Mohammed Tawfiq Abu Aisheh, Faris Abushamma

**Affiliations:** 1 Department of Urology, An-Najah National University Hospital, Nablus, PSE; 2 Department of Urology, Rafidia Surgical Hospital, Nablus, PSE; 3 Department of Radiology, An-Najah National University Hospital, Nablus, PSE; 4 Department of Medicine, Faculty of Medicine and Allied Medical Sciences, An-Najah National University, Nablus, PSE

**Keywords:** adolescent health, artificial intelligence in medicine, chatgpt, large language models, testicular torsion, urology emergency

## Abstract

Testicular torsion is a time-critical urological emergency in which every hour of delay erodes the chance of testicular salvage. Adolescents frequently delay presentation because of embarrassment, denial, and parental “watch-and-wait” behavior. We report the case of a 15-year-old boy who developed sudden, recurrent testicular pain while alone at home and, instead of waiting, queried ChatGPT for advice. The chatbot unambiguously directed him to seek urgent medical help. He presented to the emergency department within approximately two hours, underwent emergency scrotal exploration with successful detorsion and orchidopexy, and the testis was salvaged. This case illustrates an unexpected and emerging role of generative artificial intelligence as an accessible, 24/7 lay counsellor in time-critical surgical emergencies.

## Introduction

Testicular torsion is the archetypal urological emergency of adolescence, with peak incidence between 12 and 18 years [1]. The outcome is extremely time-dependent; surgical exploration within six hours of symptom onset salvages the testis in over 90% of cases, falling to roughly 50% beyond 12 hours and below 10% after 24 hours [1,2]. Despite this, large case series consistently report overall salvage rates of only 41-46%, with delayed presentation, not misdiagnosis, identified as the principal driver of testicular loss, accounting for an estimated 58% of orchidectomies [3,4]. Qualitative work has highlighted embarrassment, denial, and parental “watch-and-wait” behavior as recurring barriers in this age group [5-7].

The release of ChatGPT in late 2022 has rapidly transformed how patients access medical information. Recent systematic reviews report ~86% diagnostic-triage accuracy for GPT-4 in emergency settings [8,9] and 97% appropriate, guideline-concordant responses to advice-seeking vignettes [10]. Patients are increasingly turning to large language models (LLMs) as accessible, nonjudgmental counselors [11,12], particularly for symptoms they feel uncomfortable discussing with family or clinicians. We describe a case in which such a consultation directly precipitated emergency presentation and salvaged an adolescent testis.

## Case presentation

A previously fit and well 15-year-old boy was at home, unaccompanied, when he developed sudden, severe pain in the right hemiscrotum, identical in character to a self-limiting episode several weeks earlier. Uncertain of the urgency and reluctant to disturb his family, he opened ChatGPT and asked what he should do. The model’s response was unambiguous: “Do not wait at home. Call an adult immediately and get urgent medical help now. If the pain is sudden, strong, one-sided, swollen, lasts more than one hour, or comes with nausea or vomiting, go to the hospital or call emergency help right away because it could be a medical emergency.”

**Figure 1:**
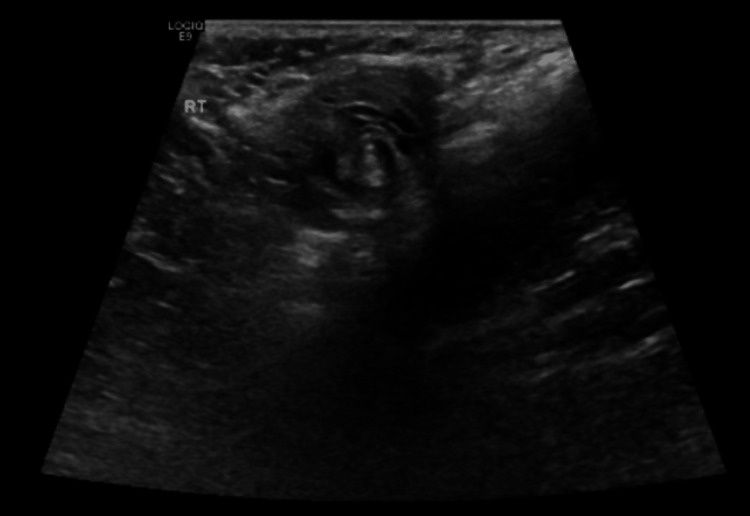
Preoperative scrotal Doppler ultrasonography displaying absent intratesticular arterial flow on the affected side, consistent with testicular torsion.

Acting on this advice, he alerted a family member and was brought to the emergency department within approximately two hours of symptom onset. On arrival, he reported pain of 9/10 with associated nausea. Examination revealed a high-riding right testis with a transverse lie, exquisite tenderness, scrotal erythema, and an absent cremasteric reflex. Preoperative scrotal Doppler ultrasonography demonstrated absent intratesticular blood flow on the affected side, consistent with testicular torsion (Figure 1).

**Figure 2:**
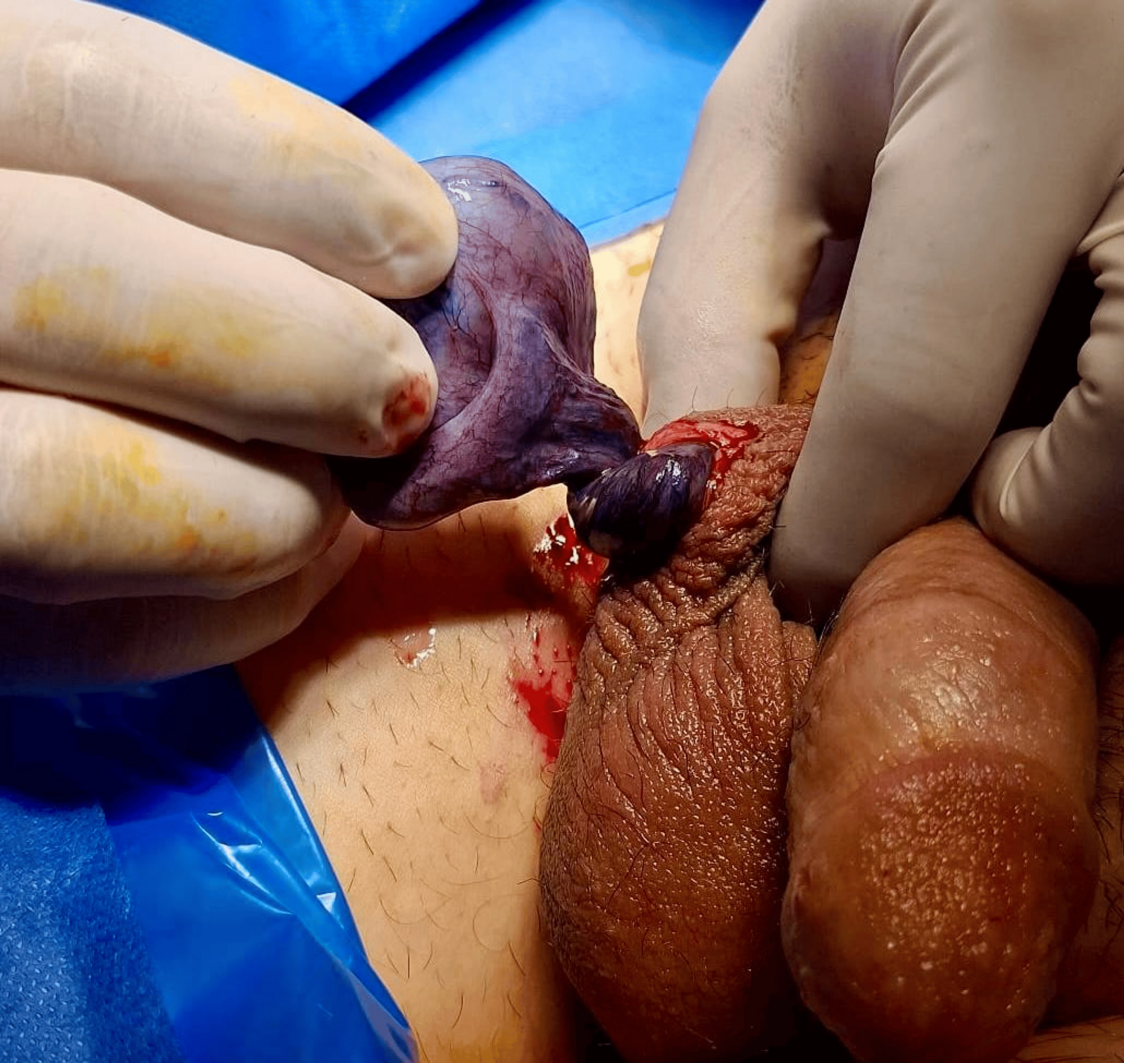
Operative scrotal exploration demonstrating torsion of the spermatic cord before detorsion.

The patient was rapidly consented and transferred to the operating theater. Scrotal exploration confirmed a 540° torsion of the spermatic cord with a viable but congested testis (Figure 2). The cord was manually detorsed, and the testis was wrapped in warm saline gauze. Following restoration of arterial pulsation and improvement in color, the testis was judged viable. Three-point orchidopexy was performed bilaterally using nonabsorbable sutures.

**Figure 3:**
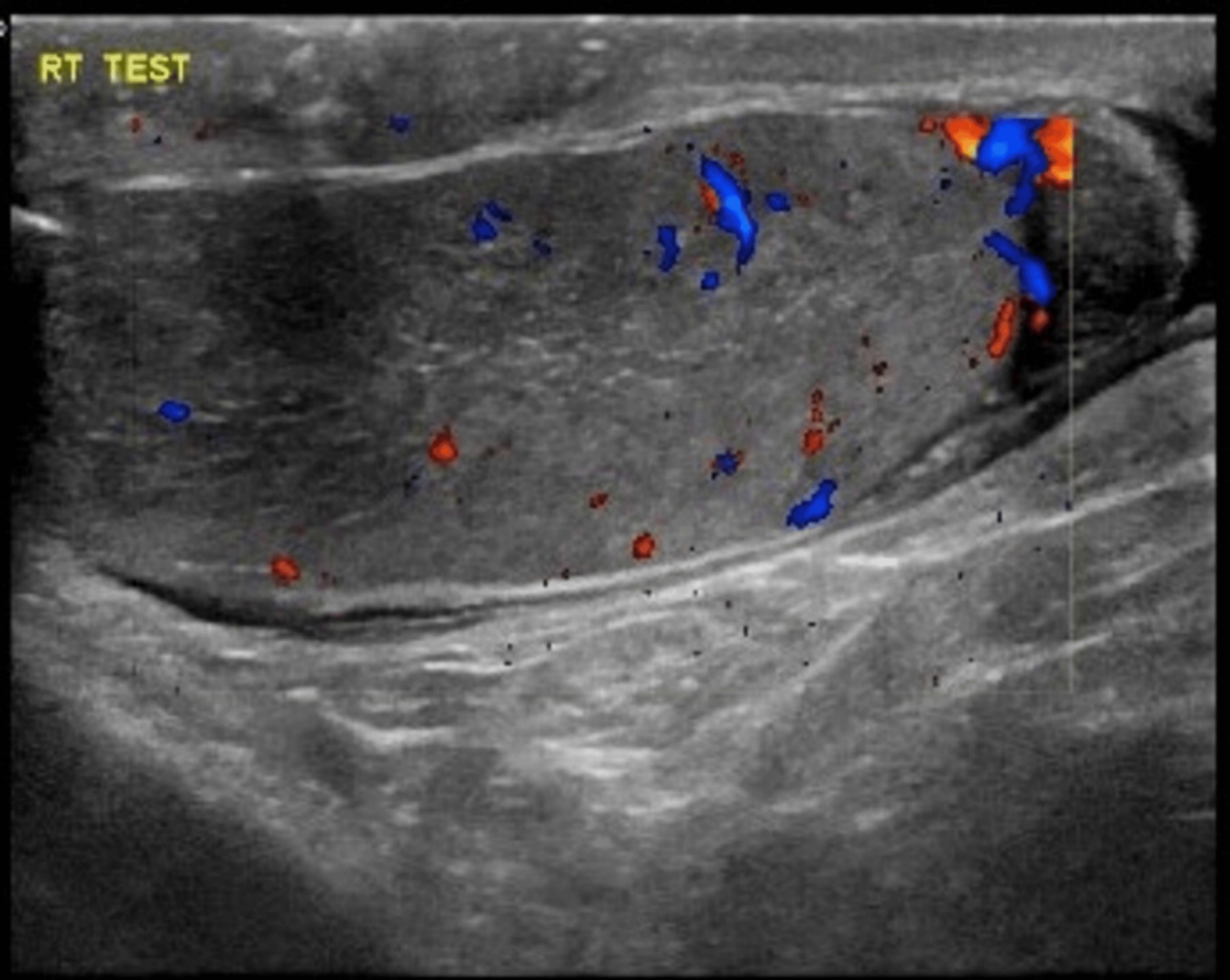
Postoperative scrotal Doppler ultrasonography displaying restored intratesticular vascularity on the affected side following detorsion and orchidopexy.

Postoperative scrotal Doppler ultrasonography on the first postoperative day demonstrated fully restored intratesticular blood flow (Figure 3). Recovery was uneventful. The patient was discharged the following morning with simple analgesia and outpatient urology follow-up.

## Discussion

This case illustrates a real-world example of generative artificial intelligence (AI) directly altering health-seeking behavior in a time-critical pediatric surgical emergency. The six-hour ischemic window for testicular torsion is well established [1-3], yet population-level salvage rates remain disappointingly low because adolescents present late [3,4]. National pediatric audit data suggest that only around 73% of boys reach the hospital within six hours, and the majority do not initially recognize the seriousness of their symptoms [2]. Qualitative studies attribute this to embarrassment, fear of “false alarms,” and the slow, parent-mediated route by which adolescents access healthcare [5-7].

LLMs are rapidly becoming a parallel, always-available channel for health information. ChatGPT-4 has shown ≥86% triage accuracy across emergency medicine validation studies [8,9,13,14] and consistently provides safety-conscious recommendations for high-acuity presentations [10,12]. In our patient, the chatbot did not act as a diagnostician but as a counselor. It dissolved the embarrassment barrier, neutralized the “watch-and-wait” temptation, and gave a clear, time-stamped instruction to seek emergency care: precisely the prompt that adolescents alone at home rarely receive.

This reframes generative AI as a public health intervention rather than a diagnostic gimmick. The same technology that has been criticized for hallucination and inequity [11,15] may, when correctly deployed for symptom counseling, shorten the interval between pain onset and definitive surgery. As LLM access becomes universal, urology and emergency medicine should consider their potential as awareness-raising adjuncts, particularly for stigma-laden conditions such as testicular pain. Important caveats remain: LLMs can hallucinate, may give inequitable advice across socioeconomic groups, and are no substitute for clinical examination [11,15]. Future work should explore how LLM responses to urgent symptoms can be standardized, audited, and integrated with formal triage pathways.

## Conclusions

In adolescent testicular torsion, where awareness and embarrassment remain the dominant determinants of testicular loss, accessible LLMs may meaningfully shorten the delay to definitive surgical care. In this 15-year-old patient, ChatGPT’s advice plausibly contributed to testicular salvage and to the preservation of future fertility, a striking real-world illustration of generative AI’s evolving role in modern medicine.

## Figures and Tables

**Figure 4:**
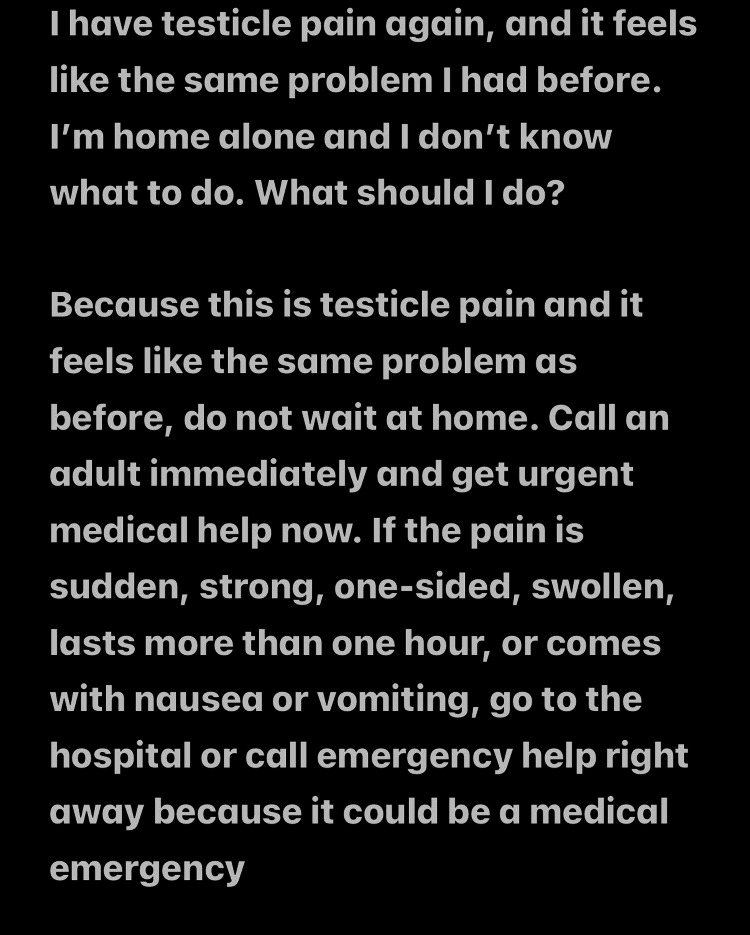
The prompt and the large language model’s response.
